# Retraction: Health risk assessment of heavy metals via consumption of dietary vegetables using wastewater for irrigation in Swabi, Khyber Pakhtunkhwa, Pakistan

**DOI:** 10.1371/journal.pone.0287203

**Published:** 2023-06-14

**Authors:** The PLOS ONE Editors

The *PLOS ONE* Editors retract this article [1] because it was identified as one of a series of submissions for which we have concerns about the integrity of the peer review process. We regret that the issues were not addressed prior to the article’s publication.

MIsrar and FMA-Z did not agree with the retraction. FA, SUR, AA, HG, MIdrees, RI, AK, and MH either did not respond directly or could not be reached.
